# The Sound of Fear: A Novel Triad of Drone, Phone, and Robot Phobia in Gaza – A Case Series

**DOI:** 10.1192/j.eurpsy.2026.11885

**Published:** 2026-07-31

**Authors:** M. Abu-Slaih

**Affiliations:** Private Clinic, Amman, Jordan

## Abstract

**Introduction:**

The psychological impact of drone warfare on civilians is known to cause significant trauma. However, the consequences of a technologically saturated battlespace, where everyday objects are weaponized, remain poorly defined. The recent war on Gaza, following precedents like the 2024 pager explosions in Lebanon, has fostered a unique environment of technological terror, leading to a novel cluster of phobic responses extending beyond drones to include personal communication devices and autonomous ground systems.

**Objectives:**

This study aims to characterize a novel triad of technology-induced phobias observed in a cohort of Palestinians in Gaza. The primary objective is to document the clinical features of a specific “technophobia” encompassing fears of drones, mobile phones, and explosive robots. We argue for the recognition of this presentation as a distinct, context-driven psychological syndrome not adequately captured by existing diagnostic categories.

**Methods:**

This case series examines 19 Palestinians (12 female; aged 15-39) in the Gaza Strip during the recent conflict. Data was collected via remote consultations (telephone, video, text messages). All patients received supportive psychotherapy. Clinical observations and patient self-reports were systematically documented and analyzed to identify common thematic and symptomatic patterns of technology-induced fear.

**Results:**

A consistent and debilitating triad of technology-related fears was identified. 1) Drone Phobia: All patients (100%) reported an intense phobic reaction to the acoustic signature of drones (the “zannana”), associated with imminent bombing and death, manifesting as full-blown panic attacks. Three children developed a specific fear of looking at the sky. 2) Phone Phobia: Approximately 80% of the cohort developed an acute fear of personal electronic devices, fearing they could explode, which triggered panic attacks. 3) Robot Phobia: Patients reported witnessing or hearing of explosive-laden ground robots, resulting in a pervasive fear of automated systems, where avoidance became a conscious survival strategy.

**Conclusions:**

The findings suggest the emergence of a complex syndrome, “War-Induced Technophobia,” characterized by a triad of fears targeting aerial, personal, and ground-based technologies. This syndrome is distinct from standard phobias as it is rooted in a realistic, continuous threat environment where everyday technology is weaponized. We argue for the critical need to recognize and classify these novel forms of trauma to develop appropriate therapeutic interventions for populations in modern, technologically advanced conflict zones.

**Disclosure of Interest:**

None Declared

